# Adjuvant Carboplatin and Paclitaxel Chemotherapy Followed by Radiotherapy in High-Risk Endometrial Cancer: A Retrospective Analysis

**DOI:** 10.1200/JGO.17.00146

**Published:** 2018-01-25

**Authors:** Renata Rodrigues da Cunha Colombo Bonadio, Renata Gondim Meira Velame Azevedo, Guilherme Harada, Samantha Cabral Severino da Costa, Vanessa Costa Miranda, Daniela de Freitas, Elias Abdo Filho, Patricia Alves de Oliveira Ferreira, Flavia Gabrielli, Maria Del Pilar Estevez Diz

**Affiliations:** All authors: Instituto do Câncer do Estado de São Paulo and Universidade de São Paulo, São Paulo, Brazil.

## Abstract

**Purpose:**

The best adjuvant treatment in high-risk endometrial cancer remains unclear. Although adjuvant chemotherapy seems to improve overall survival (OS) in locally advanced disease, the role of adding radiotherapy is not certain. We evaluated the outcomes of patients with high-risk endometrial cancer treated with adjuvant chemotherapy followed by radiotherapy.

**Patients and Methods:**

We performed a retrospective analysis of patients with high-risk endometrial cancer (endometrioid histology stages III to IVA or carcinosarcoma, clear cell, or serous histology stages I to IVA) treated with adjuvant carboplatin and paclitaxel, followed by radiotherapy, from 2010 to 2017 at a Brazilian cancer center. The Kaplan-Meier method was used for survival analysis, and prognostic factors were analyzed using the Cox proportional hazards model.

**Results:**

One hundred forty-six consecutive patients were evaluated. The OS rates were 86.2% at 3 years and 75.4% at 5 years. OS was significantly affected by pelvic lymphadenectomy (*P* = .001) and positive peritoneal cytology (*P* < .001). Three- and 5-year disease-free survival (DFS) rates were 78.3% and 69.5%, respectively. The initial site of recurrence was limited to the pelvis in 4.1% of patients, within the abdomen in 1.3%, and extra-abdominal in 11.6%. Patients with grade 1 or 2 endometrioid carcinoma had better prognosis than patients with endometrioid carcinoma grade 3 or nonendometrioid histology (3-year DFS, 93.67% *v* 68.5%, respectively; *P* = .0017).

**Conclusion:**

Adjuvant carboplatin and paclitaxel, followed by radiotherapy, is effective in high-risk endometrial cancer and associated with low rates of pelvic recurrence, which might be explained by the addition of radiotherapy. The high-risk group is heterogeneous, and the benefit of adjuvant treatment in patients with grade 1 or 2 endometrioid carcinoma is less clear.

## INTRODUCTION

Patients with locally advanced endometrial cancer (International Federation of Gynecology and Obstetrics [FIGO] stages III to IVA) and patients with nonendometrioid histologic types of endometrial cancer (regardless of stage) are included in a high-risk group. This group experiences high recurrence rates after surgical treatment and benefits from adjuvant treatment.^1,2^

In locally advanced disease, the Gynecologic Oncology Group (GOG) 122 trial showed that adjuvant chemotherapy was associated with better overall survival (OS) than whole-abdominal radiotherapy.^3^ Regarding the role of adding radiotherapy to chemotherapy in this group, preliminary results of the GOG 258 trial showed better local control with the combined therapy, but no difference in disease-free survival (DFS) was seen.^4^ Considering the available evidence, the role of sequential chemotherapy and radiotherapy in high-risk patients remains unclear.

Regarding the chemotherapy regimen, preliminary results from a phase III trial evaluating different chemotherapy regimens in the adjuvant setting were recently presented.^5^ Docetaxel plus cisplatin, paclitaxel plus carboplatin, and doxorubicin plus cisplatin were compared. There was no significant difference in OS between the three regimens (*P* = .67). Previously, in metastatic disease, a study showed that carboplatin and paclitaxel were noninferior to cisplatin, doxorubicin, and paclitaxel.^6^

The current study aims to evaluate the outcomes of patients with high-risk endometrial cancer, including endometrioid histology stages III to IVA and nonendometrioid histology stages I to IVA, treated with adjuvant chemotherapy with carboplatin and paclitaxel followed by radiotherapy.

## PATIENTS AND METHODS

### Study Design and Participants

A retrospective cohort study was done to evaluate consecutive patients with high-risk endometrial cancer treated between April 2010 and January 2017 at the Cancer Institute of São Paulo in Brazil. Patients were included if they had high-risk endometrial cancer, considered as endometrioid histology stages III to IVA or clear cell, serous, or carcinosarcoma histology stages I to IVA. These patients were treated with total hysterectomy and bilateral salpingo-oophorectomy with or without pelvic and/or para-aortic lymphadenectomy. Most of the patients underwent postoperative staging, according to the 2009 FIGO staging system. In a minority of patients who were not submitted to lymphadenectomy, preoperative images were used for staging. In accordance with 2009 FIGO staging, peritoneal cytology was not included in the staging and was not obligatory, but it was performed in a few patients. Patients were included if they had received at least one cycle of adjuvant chemotherapy and one fraction of adjuvant radiotherapy. The surveillance protocol of our institution was follow-up with clinical examination, including pelvic exam, every 3 to 6 months for 2 years and then every 6 months or annually thereafter; chest radiography annually; and CA-125 measurement at each follow-up visit if elevated at diagnosis.

Patients were excluded from the analysis if they had macroscopic residual disease after surgery or had any other malignancies within 5 years of the diagnosis of endometrial cancer (except basal cell skin carcinoma or cervical carcinoma in situ). Medical records were reviewed for demographic, clinicopathologic, and outcome information.

The study was approved by the local research ethics committee. As a result of the study being retrospective, formal consent was not required.

### Treatment

Adjuvant chemotherapy consisted of carboplatin (area under the curve, 5) and paclitaxel (175 mg/m^2^) every 3 weeks for six cycles. Granulopoiesis-stimulating factors were not used for primary prophylaxis.

This was followed by conformal external-beam radiotherapy to pelvic fields with 45 to 54 Gy, using conventional fractionation, plus weekly vaginal brachytherapy with 20 Gy in four fractions. Radiotherapy to para-aortic fields was also performed in the case of para-aortic lymph nodes metastases. Intensity-modulated radiation therapy was allowed.

### Statistical Analysis

The absolute and relative frequencies of demographic data and patient characteristics were tabulated for the analysis. Descriptive statistics were used to describe demographic patient characteristics.

OS was defined as the time from initiation of chemotherapy until death from any cause. For DFS, events were considered recurrence or death from any cause. Patients without these events were censored at the time of last follow-up.

The Kaplan-Meier method was used to calculate the OS and DFS rates, and the log-rank test was used to evaluate the difference between the curves. Univariable and multivariable analyses were performed using the Cox model to evaluate prognostic factors. The analyzed variables were age, Eastern Cooperative Oncology Group performance status, histology, FIGO stage, histologic grade, pelvic lymphadenectomy, para-aortic lymphadenectomy, brachytherapy, and peritoneal cytology. Variables that resulted in *P* < .1 were inserted in the multivariable analysis.

*P* < .05 was considered statistically significant. Statistical analysis was performed using Stata software, version 14 (StataCorp, College Station, TX).

## RESULTS

Two hundred thirty-six consecutive patients with localized endometrial cancer treated with adjuvant carboplatin and paclitaxel were identified. Patients were excluded from the analysis for the following reasons: endometrioid histology stage I or II (n = 47), residual disease after surgery (n = 16), a different treatment sequencing (n = 11), contraindication or refusal to undergo radiotherapy (n = 7), other active malignancy (n = 7), or lost to follow-up at the beginning of chemotherapy (n = 2). A total of 146 patients met our inclusion criteria and were included in the analysis.

Median age was 62 years (range, 35 to 81 years). Most of the patients had Eastern Cooperative Oncology Group performance status of 0 or 1 (98.6%), endometrioid (50.7%) or serous histology (28%), grade 3 tumor (53.4%), and FIGO stage III disease (73.3%). Only two patients had FIGO stage IVA disease, both of whom presented bowel involvement that was completely resected. Median follow-up was 29.5 months. Table 1 lists the baseline characteristics of the patients.

**Table 1:**
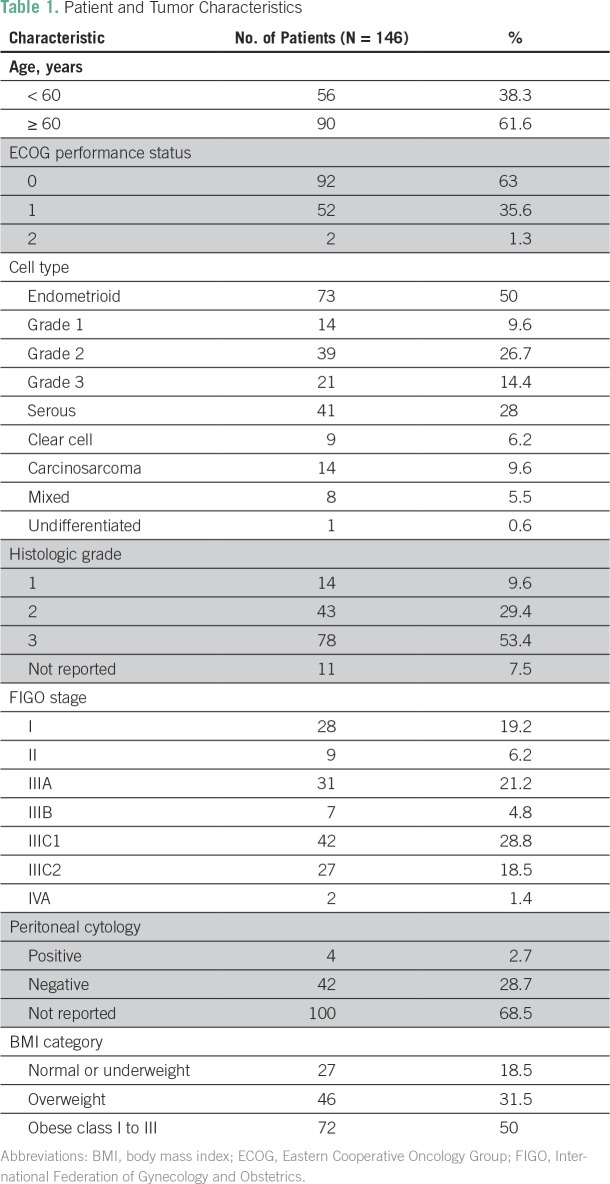
Patient and Tumor Characteristics

The majority of patients received both pelvic (90.41%) and para-aortic (82.19%) lymphadenectomy. There was good adherence to the proposed adjuvant treatment, with 84.9% of the patients completing chemotherapy and 95.2% completing radiotherapy.

**Fig 1:**
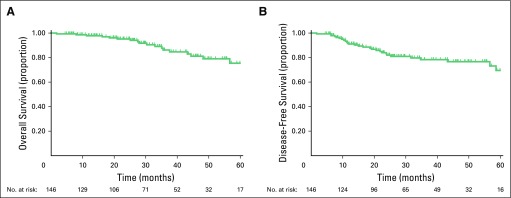
(A) Five-year overall survival. (B) Five-year disease-free survival.

Median OS and DFS were not reached. Figure 1 shows the OS and DFS curves. Nineteen deaths occurred during follow-up. The estimated 3- and 5-year OS rates were 86.2% (95% CI, 77% to 91.9%) and 75.4% (95% CI, 61.8% to 84.8%), respectively. In relation to DFS, 28 events occurred, including 25 recurrences and three deaths without recurrence. The estimated 3- and 5-year DFS rates were 78.3% (95% CI, 69.3% to 85%) and 69.5% (95% CI, 55.6% to 79.7%), respectively. The initial site of recurrence was limited to the pelvis in 4.1% of patients, within the abdomen in 1.3%, and extra-abdominal or hepatic in 11.6%.

In the univariable analysis, variables associated with OS were FIGO stage, pelvic lymphadenectomy, and positive peritoneal cytology. In the multivariable analysis, pelvic lymphadenectomy and positive peritoneal cytology remained significantly associated with OS (pelvic lymphadenectomy, yes *v* no: hazard ratio [HR], 0.15; 95% CI, 0.05 to 0.44, *P* = .001; peritoneal cytology, positive *v* negative: HR, 18.78; 95% CI, 4.70 to 74.90; *P* < .001). Variables associated with DFS in the univariable analysis were histologic grade, pelvic lymphadenectomy, and positive peritoneal cytology. Only pelvic lymphadenectomy and positive peritoneal cytology remained significantly associated with DFS in the multivariable analysis (pelvic lymphadenectomy, yes *v* no: HR, 0.31; 95% CI, 0.11 to 0.85; *P* = .023; peritoneal cytology, positive *v* negative: HR, 5.55; 95% CI, 1.58 to 19.52; *P* = .008). Table 2 lists the results of the univariable and multivariable analyses of prognostic factors.

**Table 2:**
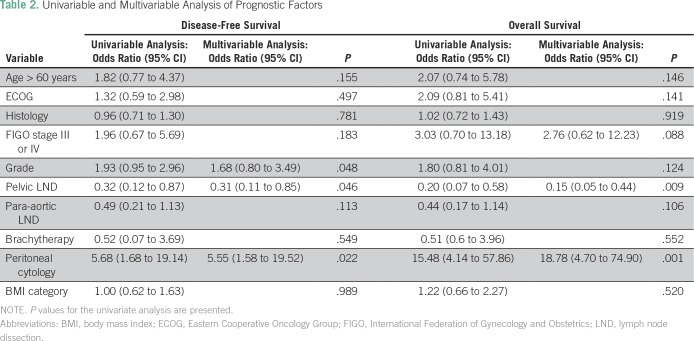
Univariable and Multivariable Analysis of Prognostic Factors

**Fig 2:**
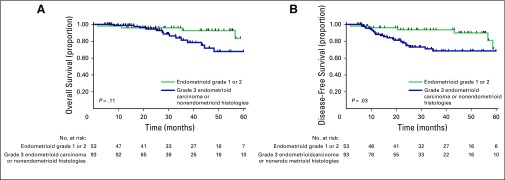
(A) Five-year overall survival and (B) disease-free survival for patients with grade 1 or 2 endometrioid carcinoma versus patients with grade 3 endometrioid carcinoma or nonendometrioid histologies.

Because patients with endometrioid histology grade 1 or 2 are expected to have a better prognosis, we performed a subgroup analysis evaluating this subgroup versus patients with endometrioid carcinoma grade 3 or nonendometrioid histologies. There was a statistically significant difference between the groups in relation to DFS (*P* = .034), but not in terms of OS (*P* = .11; Fig 2). The 3-year DFS rate was 93.67% (95% CI, 81.5% to 97.9%) for patients with endometrioid carcinoma grade 1 or 2, compared with 68.5% (95% CI, 55.5% to 78.4%) for patients with endometrioid carcinoma grade 3 or nonendometrioid histologies (HR, 4.98; 95% CI, 1.49 to 16.67; *P* = .0017); in these same patients, the 3-year OS rates were 92.67% (95% CI, 78.9% to 97.7%) and 81.3% (95% CI, 67.3% to 89.7%), respectively (HR, 2.38; 95% CI, 0.65 to 8.67; *P* = .19).

Grade 3 or 4 adverse events related to chemotherapy occurred in 47% of the patients, which were mainly hematologic (43%) and manageable. Thirty percent of the patients experienced grade 3 or 4 neutropenia, and 4.2% had grade 3 or 4 thrombocytopenia. Despite the high frequency of neutropenia, only three patients had febrile neutropenia. The second most common grade 3 or 4 adverse event was neuropathy (4.2%). Only four patients needed hospitalization as a result of toxicities. Treatment was well tolerated overall. The chemotherapy and radiotherapy acute adverse events are listed in Tables 3 and 4, respectively. Late adverse events were mainly grade 1 and are listed in Table 5.

**Table 3:**
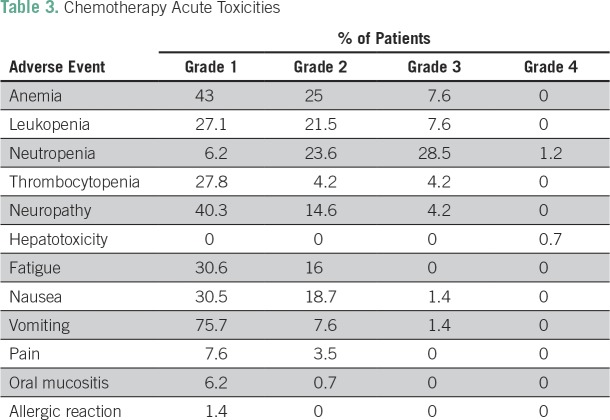
Chemotherapy Acute Toxicities

**Table 4:**
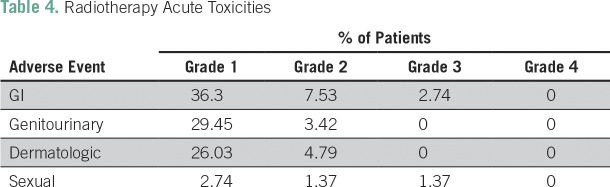
Radiotherapy Acute Toxicities

**Table 5:**
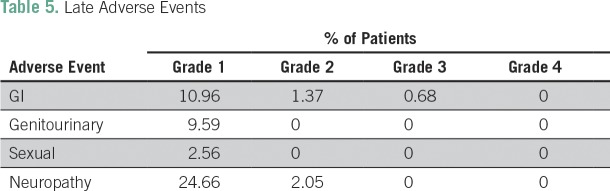
Late Adverse Events

## DISCUSSION

Our study population was similar to the one in the GOG 122 trial that compared adjuvant cisplatin and doxorubicin versus whole-abdominal radiotherapy in stage III or IV endometrial cancer.^3^ In the GOG 122 trial, the 5-year OS and progression-free survival rates were 55% and 50%, respectively, in the chemotherapy group and 42% and 38%, respectively, in the radiotherapy group. In a historical comparison, the 5-year OS and DFS rates were 75.4% and 69.5%, respectively, in our study, showing better results than those in the isolated chemotherapy or radiotherapy arms of GOG 122.

Regarding the initial site of recurrence, in the GOG 122 trial, 13% of recurrences were limited to the pelvis, 16% were within the abdomen, and 22% were extra abdominal or hepatic in the radiotherapy arm, whereas 18%, 14%, and 18% of recurrences occurred in the pelvis, abdomen, or extra-abdominal or hepatic sites, respectively, in the chemotherapy arm. In our study, recurrences were limited to the pelvis in only 4.1% of patients and were within the abdomen in 1.3% of patients. It is suggested that although chemotherapy reduces distant recurrence, radiotherapy improves locoregional control.^7^ The addition of radiotherapy to chemotherapy might explain the low rates of recurrence limited to the pelvis found in our study.

Preliminary results of the GOG 258 trial were recently presented and confirmed the improvement of local control with the addition of radiotherapy to adjuvant chemotherapy.^4^ This trial compared radiotherapy concomitant with cisplatin, followed by four cycles of carboplatin and paclitaxel, versus six cycles of carboplatin and paclitaxel. Patients with stage III to IVA disease (with < 2 cm residual disease) or stage I or II serous or clear cell uterine cancer with positive peritoneal cytology were included. Vaginal recurrence rates were 3% with chemotherapy plus radiotherapy compared with 7% with isolated chemotherapy (HR, 0.36; 95% CI, 0.16 to 0.82), and pelvic or para-aortic recurrences rates were 10% compared with 21%, respectively (HR, 0.43; 95% CI, 0.28 to 0.66). However, distant recurrence was higher in the chemotherapy plus radiotherapy group (28% *v* 21% with chemotherapy alone; HR, 1.36; 95% CI, 1 to 1.86), which is possibly explained by the lower dose of adjuvant carboplatin and paclitaxel received in the experimental arm. No difference was observed in terms of recurrence-free survival between the treatment arms. Interestingly, the 5-year OS rates in both arms (70% with chemoradiotherapy and 73% with isolated chemotherapy) were similar to that observed in our study (75.4%). These results reinforce the role of adjuvant chemotherapy as the complementary treatment modality that is responsible for the improvement in OS in high-risk endometrial carcinoma.

One important issue is that the high-risk group is heterogeneous, and patients included in this category diverge in different studies. In a recent European Society for Medical Oncology–European Society of Gynecological Oncology–European Society for Radiotherapy and Oncology consensus, the high-risk category also included stage I endometrioid carcinoma with grade 3 histology and > 50% myometrial invasion and stage II endometrioid carcinoma.^2^ These patients were included in studies evaluating chemotherapy and radiotherapy versus isolated radiotherapy.

The Randomized Trial of Radiation Therapy With or Without Chemotherapy for Endometrial Cancer (PORTEC-3) evaluated the same experimental regimen of GOG 258 (radiotherapy concomitant with cisplatin, followed by four cycles of carboplatin and paclitaxel), compared with radiotherapy in patients with stage I to III high-risk endometrial cancer.^8^ Preliminary results showed no significant difference in terms of failure-free survival (FFS) or OS. These results differ from those of the combined analysis of Nordic Society of Gynecologic Oncology/European Organisation for the Research and Treatment of Cancer and Gynaecological Oncology group at the Mario Negri Institute (MaNGO) ILIADE III trials that also evaluated stage I to III high-risk endometrial cancer and showed higher DFS (HR, 0.64; 95% CI, 0.41 to 0.99; *P* = .04) and cancer-specific survival (HR, 0.55; 95% CI, 0.35 to 0.88; *P* = .01) with sequential chemotherapy and radiotherapy versus isolated radiotherapy.^9^ In a subgroup analysis of PORTEC-3, stage III endometrial carcinoma presented a benefit in FFS with the addition of adjuvant chemotherapy (5-year FFS, 69.3% *v* 58% with radiotherapy alone; HR, 0.66; 95% CI, 0.45 to 0.97, *P* = 0 =.032). This subgroup (stage III endometrial carcinoma) also represents the majority of the population in our study, and once again, the results observed in the adjuvant chemotherapy and radiotherapy arm are consistent with ours (5-year DFS rate of 69.5% in the current study).

In GOG 258 and PORTEC-3 trials, the experimental arm evaluated cisplatin concomitant with radiotherapy, followed by sequential carboplatin and paclitaxel. With this strategy, it is difficult to clarify whether both concomitant and sequential chemotherapy are in fact necessary. Moreover, the better sequence between adjuvant chemotherapy and radiotherapy is still unknown. Because chemotherapy is the treatment associated with improvement in survival in locally advanced disease, it is performed before radiotherapy in our institute. We hypothesized that tolerability to chemotherapy is better when given before radiotherapy, improving chemotherapy completion. Our results showed that 84.9% of the patients completed chemotherapy with this strategy. In the PORTEC-3 trial, 80% of the patients completed carboplatin and 72% completed paclitaxel, which might be explained by lower tolerability to chemotherapy when it is given after radiotherapy concomitant with cisplatin.^8^ In GOG 258, 83.7% of the patients completed four cycles of carboplatin (completion of paclitaxel is not reported).^4^

Another important point is that our treatment included both pelvic radiotherapy and vaginal brachytherapy, according to our institute protocol at the time. However, a recent American Society for Radiation Oncology guideline suggested that because data are lacking to validate vaginal brachytherapy in addition to pelvic radiotherapy, the combination of the two modalities should be reserved for patients with risk factors for vaginal recurrences.^10^ In early-stage endometrial cancer with high- or intermediate-risk features, the PORTEC-2 trial showed that vaginal brachytherapy is noninferior to pelvic radiotherapy^11^ and can be considered the treatment of choice for these patients. For women with high-risk early-stage (endometrioid adenocarcinoma stage IB grade 3 or stage II endometrioid and any stage nonendometrioid histologies) or locally advanced disease (stages III to IVA), pelvic radiotherapy is usually recommended, despite the fact that there is no high-level evidence to support it.^10,12^

Finally, as shown in the studies mentioned earlier, the high-risk group include patients who might benefit differently from the adjuvant treatment strategies. In our subgroup analysis, patients with grade 1 or 2 locally advanced endometrioid carcinoma had better prognosis than patients with grade 3 endometrioid carcinoma or nonendometrioid histologies. Despite being in the same risk group, patients with grade 1 or 2 endometrioid carcinoma might not derive as much benefit as others from adjuvant therapy.

Our study has limitations as a result of the risk of bias from retrospective studies. Furthermore, many statistical tests were performed, which increases the risk of false-positive results. However, regardless of these limitations, we believe that our study has value as a hypothesis-generating study.

In conclusion, our data suggest that adjuvant carboplatin and paclitaxel, followed by radiotherapy, in this particular group of patients with high-risk endometrial cancer (endometrioid histology stage III to IVA and nonendometrioid histology stage I to IVA) is safe and effective. The rates of recurrence limited to the pelvis were low, probably because of the addition of radiotherapy to chemotherapy. Long-term results of the ongoing studies are still awaited to define the best adjuvant strategy.
